# Challenges and opportunities for general practice specific CME in Europe – a narrative review of seven countries

**DOI:** 10.1186/s12909-022-03832-7

**Published:** 2022-11-07

**Authors:** Christin Löffler, Attila Altiner, Sandra Blumenthal, Pascale Bruno, An De Sutter, Bart J. De Vos, Geert-Jan Dinant, Martin Duerden, Brigitte Dunais, Günther Egidi, Bernhard Gibis, Hasse Melbye, Frederic Rouquier, Thomas Rosemann, Pia Touboul-Lundgren, Gregor Feldmeier

**Affiliations:** 1 Institute of General Practice, Rostock University Medical Center, Doberaner Str. 142, 18057 Rostock, Germany; 2 Institute of General Practice and Family Medicine, Charité - Universitätsmedizin Berlin, Berlin, Germany; 3 Speaker section advanced training German Society for General Practice and Family Medicine (DEGAM), Berlin, Germany; 4 Centre Hospitalier Universitaire de Nice, Département de Santé Publique, Université Côte d’Azur, Nice, France; 5 Department of Public Health and Primary Care, Centre for Family Medicine, Ghent University, Ghent, Belgium; 6 National Institute for Health and Disability Insurance, Brussels, Belgium; 7 School for Public Health and Primary Care, Faculty of Health, Medicine and Life Sciences, Maastricht University, Maastricht, The Netherlands; 8 Centre for Medical Education, Cardiff University, Cardiff, UK; 9 General Practice Bremen Germany, Deputy Speaker section advanced training German Society for General Practice and family medicine (DEGAM), Bremen, Germany; 10 National Association of Statutory Health Insurance Physicians (KBV), Berlin, Germany; 11 General Practice Research Unit, UIT the Arctic University of Norway, Tromsø, Norway; 12 General Practice, St Cezaire sur Siagne, France; 13 Institute of Primary Care, University of Zurich, Zurich, Switzerland

**Keywords:** Continuing medical education, General practice, Narrative review, Curriculum, Program evaluation

## Abstract

**Background:**

Several changes have led to general practitioners (GPs) working in a more differentiated setting today and being supported by other health professions. As practice changes, primary care specific continuing medical education (CME) may also need to adapt. By comparing different primary care specific CME approaches for GPs across Europe, we aim at identifying challenges and opportunities for future development.

**Methods:**

Narrative review assessing, analysing and comparing CME programs for general practitioners across different north-western European countries (UK, Norway, the Netherlands, Belgium (Flanders), Germany, Switzerland, and France). Templates containing detailed items across seven dimensions of country-specific CME were developed and used. These dimensions are role of primary care within the health system, legal regulations regarding CME, published aims of CME, actual content of CME, operationalisation, funding and sponsorship, and evaluation.

**Results:**

General practice specific CME in the countries under consideration are presented and comparatively analysed based on the dimensions defined in advance. This shows that each of the countries examined has different strengths and weaknesses. A clear pioneer cannot be identified. Nevertheless, numerous impulses for optimising future GP training systems can be derived from the examples presented.

**Conclusions:**

Independent of country specific CME programs several fields of potential action were identified: the development of curriculum objectives for GPs, the promotion of innovative teaching and learning formats, the use of synergies in specialist GP training and CME, the creation of accessible yet comprehensive learning platforms, the establishment of clear rules for sponsorship, the development of new financing models, the promotion of fair competition between CME providers, and scientifically based evaluation.

## Background

In recent years, general practice has experienced important changes and new developments in various European countries. This regards, for instance, the differentiation of competences of general practitioners (GPs), the cooperation within primary care and with different providers of secondary care, the delegation of tasks as well as the provision of patient information [1, 2]. Also, epidemiological developments such as population aging and the increase in the number of patients with chronic diseases and multimorbidity pose a challenge to general practice [3]. The latter, as well as the increasing demands of rapid changes in medical knowledge and the demands of authorities and patients for quality and safety of medical treatments, contribute to the current and future challenges of the GP’s daily work and are important drivers for continuing medical education (CME). Along with these developments, general practice has emancipated itself. Today, general practice is no longer understood as a conglomerate of different medical subjects, but as an independent speciality with a specific way of working. As GPs are concerned with health conditions in a low-prevalence setting, this includes, for instance, procedures that are different from specialised medicine [4].

As general practice has changed, so has CME of general practitioners: Today, based on the findings of research into learning styles, medical education is increasingly moving from the traditional teacher-centred approach towards a learner-centred approach. Whereas in the past the focus of academic and post-academic education was solely on the cognitive level of imparting knowledge, today the acquisition of competences, performance in real treatment situations and the development of a professional attitude are essential [5, 6]. This paradigm shift is increasingly reflected in the education, specialist training and CME of todays and future GPs. Nonetheless, the way CME for GPs has developed, varies considerably across countries and health systems.

In this narrative review we aim to identify the potential for future improvement of general practice specific CME by comparing CME development in an international perspective.

## Methods

### Study design and setting

General practice continuing medical education programs across different European countries are assessed, analysed, and compared. We thereby focus on the United Kingdom of Great Britain and Northern Ireland (the UK), Norway, the Netherlands, Belgium (Flanders), Germany, Switzerland, and France. The selection of countries considered in this international comparison aimed to analyse general practice continuing medical education in different health care systems in Europe and to show both different and similar developments. However, in order to derive insights from best practice examples, we have focused on countries that for the most part already have a more or less elaborated CME system. This resulted in a focus on north-western European countries.

### Data selection

As a first step, for each country local experts in the field of primary care specific CME training for GPs we contacted (MD for the UK, HM for Norway, GJD for the Netherlands, ADS and BDV for Belgium, AA, SB and GE for Germany, TR for Switzerland and BD, FR, PB, and PTL for France). These experts were characterised by the fact that they had acquired specific knowledge about the respective country-specific GP training system over many years as academic general practitioners or as experts of corresponding specialist institutions. For each country, experts performed literature searches for regulations and formats for general practice continuing education. In addition to human medicine literature databases such as Pubmed, grey literature (writings of country-specific institutions and associations, congress reports, academic and non-academic writings, etc.) were critically assessed. Since structures, institutions, associations as well as the way information is published differ greatly from country to country, the experts involved took different approaches to obtaining information depending on the country-specific context. In some countries, for example, responsible representatives were also contacted personally.

### Data analyses

Subsequently, to analyse CME programs we developed templates containing detailed items across seven dimensions of country-specific CME. Following the approach in qualitative studies, these dimensions were partly defined deductively in advance (top-down), partly derived inductively from the material studied (bottom-up) [7, 8]. These dimensions are: the role of primary care within the health system, legal regulations, published aims of CME, actual content of CME, operationalisation, funding and sponsorship, and evaluation. We also took developments over time into account. Also, in preparation of the country-specific reports we used qualitative semi-structured expert interviews with country experts to gain additional insight [9–11]. These were conducted with four of the country experts involved through video conferences. The interview guideline was based on the deductively established dimensions and was expanded during the process to include new dimensions [7, 8]. The interviews had a length of 120–230 minutes. Both the preparation of the country reports by experts and the conduct of the interviews were circular, with unresolved or emerging issues being clarified in an iterative process [12]. To analyse data a thematic approach was taken, and existing data was triangulated for each country. Based on this in-depth information on country-specific CME for GPs we synthesised the material and derived tables presenting information on a meta-level. The results reflect the outcome of the literature research and are based entirely on it. Personal assessments of the experts/authors are reflected in the discussion. Due to the design of the study, ethical approval was not required.

## Results

In Tables 1, 2, 3, 4, 5, 6 and 7 we, first, present country-specific information for the above stated dimensions. Next, we compare dimensions across countries.

**Table 1:** CME for GPs in the UK

**Role of primary care within the health system**	The UK National Health Service (NHS) has been funded by taxes since 1948. The use of a medical consultation, investigation and medicines is free of charge for citizens. Patients are registered via lists with practices for health care. GPs act as gatekeepers, i.e. patients usually go to their practice before a decision to refer them to a specialist or to hospital. GPs manage most diseases, including complex chronic diseases.
**Legal regulations**	The General Medical Council (GMC) compiles and keeps up to date a list of all licensed doctors in a region (Performers List). Since 2013, a prerequisite for remaining on this list and thus being able to practise as a doctor is proof of participation in Continuing Professional Development (CPD), which is provided in the form of a portfolio [13]. This is submitted annually and discussed with a peer (the ‘appraiser’) during an appraisal. The doctor is recertified (‘revalidated’) by the GMC every 5 years on the basis of satisfactory completed appraisals.
**Published aims**	Doctors are expected to complete a wide range of CPD activities [14]. The aim is to ensure that all doctors work according to the latest medical standards and knowledge and that patient care is safe. In this way, doctors are expected to refresh and expand existing knowledge, acquire new knowledge and skills, and reflect on societal changes that affect their daily professional lives.
**Actual content**	Specific learning objectives are not set; rather, it is up to the GPs to draw up a plan aimed at their personal professional development, supported by their appraiser. This plan serves in orientation for further training in the following years. In addition to clinical topics, topics such as research, teaching, training and practice management may also be included. As well as conferences and courses, CPD also includes audit, reading, and online research. Around 50 credits (50 hours) are expected annually [15].
**Operationalisation**	Since 2013, Responsible Officers have been assigned to all GPs to guide the CPD process [16]. Responsible Officers act as a link between appraisers, GPs and the GMC and make recommendations about the recertification of GPs. The GMC is responsible for issuing a national framework for recertification and making decisions regarding the recertification of all doctors.
**Funding and sponsorship**	The GMC is financed by contributions from doctors. GPs often meet the costs of their CPD themselves. GPs from Wales and Northern Ireland receive an annual allowance towards appraisal of 300 ₤; those in England and Scotland receive between 200 and 500 ₤ depending on the region. Pharmaceutical companies also organise and fund training events for general practitioners. In 2007, the pharmaceutical industry financed about half of all training events, including travel and accommodation [17], and this proportion has subsequently increased.
**Evaluation**	Since all activities - from reading to professional interaction and conferences - are part of CPD, there is little critical examination of the learning content; nor is the benefit in terms of health care aspects formally evaluated [18]. Appraisers are expected to assess the content and advise the Responsible Officer if it appears inadequate. Many events organised by pharmaceutical companies do not require formal approval and the programme is not controlled in terms of content, speakers, or advertising content (although companies are expected to adhere to a “code of conduct”).

**Table 2:** CME for GPs in the Netherlands

**Role of primary care within the health system**	Patients are assigned to a GP of their choice via lists. GPs often work in general practice centres where they, together with other health professionals, care for the complete spectrum of diseases varying from all kinds of acute conditions to chronic diseases. These centres are usually run by the GPs themselves. Referrals to specialists are made in the case of unforeseen courses of disease or for diagnostic or treatment difficulties [19].
**Legal regulations**	GPs are centrally registered on a national list. Only registered doctors can practise their profession. Recertification takes place every 5 years. This requires, among other things, proof of CME participation. These must comprise 40 CME credits per year, i.e., 200 CME credits in 5 years. In addition, doctors can also obtain continuing education credits through teaching and coaching in the clinical field as well as through publications or their own dissertation [1, 20].
**Published aims**	The goals of general practice training are formulated rather superficially and focus on the fact that GPs should generally continue their education to be aware of the current state of research. This inevitably leaves a lot of room for different interpretations.
**Actual content**	There is no CME curriculum that prescribes subject areas. All GPs are free to choose their own topics for CME training. At the same time, thematic interests are often queried at the end of events, so that they have an influence on future offers [1].
**Operationalisation**	CME training can be offered by practically any institution/person. Accreditation is carried out by means of the Gemeenschappelijke Accreditatie Internet Applicatie (GAIA) and considers, among other things, the topic, learning objectives, content, and teaching material. General practice training is usually offered and conducted by GPs. If necessary, locally active experts, such as specialists or pharmacists, are invited.
**Funding and sponsorship**	As training is usually offered within networks of doctors, there are no or only extremely low costs. For about 20 years there is a decrease in the influence of pharmaceutical companies on (general) practitioner training. Today, there are no more training courses that are linked to pharmaceutical companies in terms of content or organisation. This is regularly and strictly controlled.
**Evaluation**	Training is evaluated by the participants. A standardised template is used for this purpose. This short evaluation is then given to local employees of the Gemeenschappelijke Accreditatie Internet Applicatie (GAIA). Based on the evaluation, they confirm the successful implementation of the training. An evaluation that would verify the learning success of the participants or lead to an increase in the quality of care does not take place [21–23].

**Table 3:** CME for GPs in Norway

**Role of primary care within the health system**	General practice is the basis of health care and GPs act as gatekeepers: without a referral from them, patients cannot see specialist doctors (except private and more expensive specialists in some cities). Patients are assigned to GPs via lists. On average, a GP sees 1100 patients. Many GPs take over community health care tasks 1 day a week [24, 25].
**Legal regulations**	Norway uses a recertification system: specialists in general practice must be recertified every 5 years. Although they can no longer be deprived of their specialist title as of 2019, they can only bill consultations at a reduced rate if they fail to provide required evidence, e.g., with regard to further training [26, 27].
**Published aims**	The aim of CME in general practice is to ensure that GPs are committed to lifelong learning. In contrast to CME training, the list of learning objectives in specialized education to become a general practitioner is very long and also includes attitudes, skills and reflection on medical practice. There are no such clearly defined learning objectives for CME training.
**Actual content**	GPs must plan about 40 days per 5-year period for CME activities. The following training activities are obligatory: 100 CME credits from courses on six topics. One course should be on acute medicine (including resuscitation). 20 CME credits through 2 full-day practice visits to (or from) another GP practice. 20 CME credits through regular participation in a general practice CME group. A further 160 credits are made up of freely selectable courses and a comprehensive list of continuing education activities (teaching, research, etc.).
**Operationalisation**	Most of the training is organised or supported by the Norwegian Medical Association. The Norwegian General Medical Association is entrusted with quality assurance. All continuing education must be approved by a specialty-specific committee of the Norwegian Medical Association. Each specialty - including general practice - has its own committee. The training programme is submitted to this committee, which approves or rejects it. CME training and specialized education to become a GP is often combined [28].
**Funding and sponsorship**	Most of the costs of training in Norway are covered by the Norwegian Medical Association’s training fund. As a rule, GPs have to pay the course fees for the training themselves but may be reimbursed travel expenses. Profit-oriented training companies play only a minor role. Although pharmaceutical companies organise training for GPs, they do not contribute CME credits for recertification.
**Evaluation**	Norwegian Medical Association training courses are evaluated by the participants. Approved online courses usually end with a knowledge test. After practice visits, GPs must write a report. In the course of the new regulations on general practice continuing education and training issued in 2019, the evaluation of continuing education has also been put into focus.

**Table 4:** CME for GPs in Belgium (Flanders)

**Role of primary care within the health system**	There is no compulsory registration of patients with a general practitioner. Patients can see specialists or the hospital for treatment without a referral. Payment is mostly on a fee-for-service basis. Only in a minority of practices is there a patient list in the sense of an enrolment. Nevertheless, there has been some strengthening of general practice in recent decades, e.g., patients have recently been financially incentivised to specify a GP as the manager of their “global medical record” [29].
**Legal regulations**	The law of 22 April 2019 on good quality practice in health care applies to all health care providers. According to this law, a health professional may only provide health services if he can prove the required skills and experience. For this purpose, he/she must “keep the necessary data, preferably in electronic form, showing that he/she has the required skills and experience” [30, 31].
**Published aims**	CME in Belgium is strongly linked to the development of the accreditation system. This was explicitly created to improve the quality of care through CME on the one hand and to optimise cost efficiency in the health system on the other.
**Actual content**	In Belgium, doctors are free to choose the content and provider of their CME training. This is because they are considered to be in the best position to assess their own training needs. To this end, they can attend any accredited continuing education course. Each doctor is expected to earn 20 credits per 12-months accreditation period. However, there are two exceptions: Doctors should specifically address the topics of ethics and economics (at least 3 credits per year). Attendance at at least 2 meetings of a local quality circle is also required [32].
**Operationalisation**	There are hardly any formal requirements for general practice continuing education within (voluntary) medical accreditation. For accrediting training events (with very few exceptions), only content-related aspects such as topic and speaker are considered. Due to these regulations, there are numerous training events in Belgium that are offered by numerous organisers. All relevant information and a list of accredited training events are published online [33]. At the local level, the leaders of the respective quality circles are responsible for coordination.
**Funding and sponsorship**	Local quality circles receive limited financial support [34]. Accredited GPs receive a lump sum of 622.61 EURO as compensation for training costs within the 12-month accreditation period. In addition, accredited doctors receive a higher remuneration than non-accredited doctors. Depending on the format, the pharmaceutical industry can finance training events to a limited extent. In general, the current regulations aim to strongly limit the influence of the pharmaceutical industry.
**Evaluation**	Only for e-learning courses is there a requirement for compulsory evaluation. The evaluation of other formats is carried out by the organisers on a voluntary basis. The accreditation system itself has only been evaluated once so far, in 2003. The participating GPs believed the accreditation had improved both the quality of the training and the quality of the medical services they provided. It had also increased the number of training courses in which they had participated.

**Table 5:** CME for GPs in Germany

**Role of primary care within the health system**	Although general practice centred care is gaining in importance as a model in numerous German regions, it is not being implemented across the country. In addition to general practitioners in solo and joint practices, and medical care centres (MVZ), there are practices containing specialists in numerous disciplines. Patients can consult specialists in addition to their general practitioner, without the need for a GP referral or coordination.
**Legal regulations**	The Model Continuing Education Regulation (Musterfortbildungsordnung) of the German Medical Association (Bundesärztekammer) of 29.05.2013 regulates formal criteria for CME in Germany. The respective federal state medical associations follow these as far as possible. Since there is almost no institutional accreditation, each CME event is usually certified individually by the respective medical association. Thus, there is no uniform federal state or national system for providers of CME. Germany is largely characterised by an unregulated CME system [35].
**Published aims**	The Model Continuing Education Regulation of the German Medical Association defines the goal of CME as follows: “The continuing education of physicians serves to maintain and continuously develop professional competence in order to guarantee high-quality patient care and ensure the quality of medical professional practice” [36]. This broad definition leaves a lot of room for variation in content and structure.
**Actual content**	With 250 CME credits in 5 years, the Model Continuing Education Regulation determines the necessary scope of CME. In addition, it regulates, among other things, the evaluation and recognition of continuing education measures. While the medical associations do not have any defined learning objectives or curricula, some stakeholders have developed more or less elaborated subject matter catalogues or syllabi and formulated overarching learning objectives [35, 37].
**Operationalisation**	There is no overarching coordination of training foci or events in Germany. Rather, the German CME system can be described as a juxtaposition of numerous qualitatively very heterogeneous offers [38, 39]. Most of the existing organisations and institutions operate regionally or locally. The certification of continuing education programmes is carried out exclusively by the responsible federal state medical associations.
**Funding and sponsorship**	GPs are only incentivised to participate in further training by the award of CME points and the possibility of billing Disease Management Programmes (DMPs). Training obligations from GP contracts can present further incentives. However, this applies to a limited number of GPs. Depending on the quality, duration and sponsorship, the costs for GP training vary greatly and can hardly be averaged out. Sponsorship is still widespread in the GP training landscape in Germany, even if individual actors and initiatives categorically reject industry sponsorship [40].
**Evaluation**	CME courses are evaluated by the accrediting institutions (regional medical associations). In most cases, this is done by means of categorical questionnaires, which are filled out by the participants according to subjective aspects. Objective evaluations, which e.g., also record the learning and competence gains of the participating GPs, are hardly ever used. In addition to the evaluations of these regional medical associations, organisers also evaluate their training courses.

**Table 6:** CME for GPs in Switzerland

**Role of primary care within the health system**	The special importance of general practice for primary care was incorporated into the constitution in 2014 following a popular initiative. In Switzerland, there are a number of privately financed insurance models that link a payment reduction to the obligation to always contact the general practitioner first, except in an emergency [41].
**Legal regulations**	Since the Medical Professions Act came into force in 2007, CME has been one of the professional duties required by law. The realisation and implementation of this duty was left to the professional organisations, which created the Swiss Institute for CME (SIWF) for this purpose [42, 43].
**Published aims**	The CME training programme of 2019 does not contain a specific definition of the content or learning objectives of the trainings.
**Actual content**	Together with the Swiss Society of General Internal Medicine (SGAIM), the SIWF offers a CME training diploma: Every year, 50 credits of a structured CME training plan need to be obtained (at least 25 hours of specialist core training with a strong internal medicine focus; and a maximum of 25 hours of extended training) plus 30 credits of self-study. 1 credit corresponds to 45–60 minutes of study time [44].
**Operationalisation**	CME is regulated nationally by the SIWF. CME credits are applied for via the respective professional associations. These in turn are guided by the SIWF guidelines. Since the cantons are responsible for licensing the profession, they are also responsible for monitoring the obligation to provide CME [43].
**Funding and sponsorship**	Training is organized by doctors or a medical expert committee. Companies or service providers cannot be organisers of CME. In case of financial support, several (pharmaceutical) companies should be involved in sponsoring. Thus, ‘mono’ sponsoring is not possible. Sponsors should not exert any influence on the content, schedule or speakers. Participants shall make an appropriate contribution to the costs (participation fee plus costs for travel and accommodation) [44].
**Evaluation**	An evaluation as part of the successful completion of the course is obligatory for the awarding of credits. However, the way this is done is not controlled.

**Table 7:** CME for GPs in France

**Role of primary care within the health system**	The “médecin généraliste” plays a central role in the French health system and is the first point of contact for the population in health matters. Since 2006, every patient is called upon to register with a family doctor of his or her choice. GPs are increasingly working in group practices, sometimes with adjoining paramedical staff, but many still work alone. In order to consult a specialist, a GP referral is required in the vast majority of cases [45].
**Legal regulations**	After numerous reforms, the Agence Nationale du Développement Professionnel Continu (ANDPC) has been monitoring the implementation of priority training content for each medical specialty for 3 years at a time since 2019. A recertification system is also to be introduced in France from 2021 [46]. Among other things, CME will also be recorded and evaluated during recertification [47].
**Published aims**	According to the French Health Code, the general objectives of CME are to maintain and update medical knowledge and skills and to improve medical practice. According to the Collège de médecine Générale (CMG), GP training should include the concept of the “reflective doctor”, in which the GP reflects on his or her medical practice, compares it with medical guidelines and looks for ways to improve it [48].
**Actual content**	The respective topics and learning objectives of the training courses are jointly determined by the Ministry of Health, the national professional councils (CNP) and the national health insurance fund for a period of 3 years. For the period 2019–2022, these include for instance developmental assessment of children, care of multimorbid patients, care of high-risk patients with cardiovascular diseases, psychotherapy and mental health, prevention of social exclusion, emergency care, communication with patients and their social environment, and practical-technical skills [49, 50].
**Operationalisation**	There are many providers of GP training, both at national, regional, and local level. These must be approved by the ANDPC (which checks that the content and the various training methods comply with the guidelines of the Haute Autorité de Santé) in order for GPs to validate a training course [51, 52].
**Funding and sponsorship**	Funding for GP training is provided by the ANDPC, which had a budget of 190 million euros in 2020. Half of the budget is used to compensate GPs for lost income during the time spent on training. The other half is used to remunerate the speakers and finance the logistics. Today, the pharmaceutical industry is no longer allowed to participate in CME. Instead, the industry co-finances medical training through a special tax [53].
**Evaluation**	The ANDPC is responsible for the approval of further training. The scientific and didactic content is controlled by an independent scientific committee. Participants are often asked to fill out an evaluation questionnaire at the end of a training event. However, neither the learning outcomes nor the existing CME concepts or improvements in medical care as a whole are recorded here [53].

### The role of primary care within the health system

The UK, Norway and the Netherlands are characterised by a more or less pronounced gate-keeper system, in which patients always - except in an emergency - consult their general practitioner first when they have health problems. In these countries, patients are assigned to their GP or the corresponding practice via lists. GPs are expected to manage complex chronic conditions independently. Referrals to specialists, who work almost exclusively in hospitals, are exceptional and not the rule. GPs are usually supported in their practice by different kinds of health care professionals, such as nurses and pharmacists.

In Switzerland and France, patients are incentivised to enrol on a voluntary basis and to consult their GP first. A growing proportion of the population is opting for this type of insurance. The situation is similar in Belgium (Flanders). Although there is no compulsory registration with a GP, patients receive a higher reimbursement of their consultation costs if they are registered with a practice. Of the countries considered, Germany is the only country with a health system that grants patients largely “unhindered” access to specialists - despite regionally successful gate-keeper models. In most cases, patients with chronic illnesses are seen in parallel by their GP and by (several) specialists in private practice.

### Legal regulations

The UK, the Netherlands and Norway have a recertification system. There, GPs must fulfil several requirements every 5 years to continue working as a GP. This includes participation in further training for GPs. The UK and the Netherlands have the stricter system of recertification. In case of failure to meet specified requirements, doctors are prohibited from continuing to practise medicine. In Norway, on the other hand, failure to fulfil requirements results in reduced remuneration. In Belgium, GPs have been able to obtain de facto recertification on a voluntary basis since 1994 – though officially the term “recertification” is not used. Linked to this is the provision of CME. A growing proportion of Belgian GPs use this system. In 2021, France introduced a recertification system with a long transition period. All recertification systems are seen as quality promotion instruments, in which continuing medical education is understood as an essential component.

Switzerland and Germany have a much less regulated system of GP training. In Switzerland, GP training has been mandatory by law since 2007 and is operationalised via the corresponding professional organisation. In Germany, the Model Continuing Education Regulation of the German Medical Association has regulated the formal criteria of CME since 2013.

### Published aims of CME

In all countries considered, the objectives of general practice continuing medical education are broadly formulated. In the UK, GPs are expected to regularly review the latest scientific standards, to refresh and expand existing knowledge, to acquire new skills and to reflect on societal changes that affect their daily work. CME is largely understood as continuing professional development (CPD) centring on the learning process of the individual GP. The goals of CME are defined similarly in France. In addition, reference is made here to the concept of the reflective doctor, who reflects on her or his own actions to enter into a continuous learning process. In Germany, to maintaining and continuously developing professional competence, special reference is made to ensuring high-quality patient care and safeguarding the quality of medical professional practice. Similarly, in Belgium, CME aims at the quality of care and the cost-effectiveness of the health care system. In the Netherlands, Norway and Switzerland, the objectives of general practice CME are not described at all or only implicitly.

### Actual content of CME

In the countries considered, there are no comprehensive training curricula in the sense of longitudinal learning objectives aimed at different areas of competence, which would define the content of general practice CME. GPs in the UK, the Netherlands and Belgium have the greatest freedom in choosing topics for continuing education courses - although this freedom can also be viewed critically. While GPs in the UK independently determine the content of GP training in consultation with a GP peer (who in turn reports to a Responsible Officer) within the framework of a training portfolio, GPs in the Netherlands largely train in informal groups. They also determine the contents independently, but together. In Belgium, only a small part of the content of continuing education is determined (ethics and economics). The content specifications of general practice CME in Norway, Switzerland and Germany are rather vague. Nevertheless, there are differences. In Norway, at least one course (at least 15 hours) must deal with the topic of acute medicine. In Switzerland, a distinction is made in terms of content between subject-specific core continuing education and extended continuing education, which allows more leeway in terms of content. In each area, 25 credits (approx. 25 h) are to be earned annually. And in Germany, GPs are indirectly incentivised via the possible remuneration to attend CME courses with direct relevance for Disease Management Programs (DMP). In contrast to these different levels of content freedom of GP training, only France has a system in which, at least from 2021 onwards, the priority setting and learning objectives of general practice continuing education are to be defined and updated every 3 years. The role of the peer in continuing education is most pronounced in the UK and the Netherlands.

### Operationalisation

The organisation of general practice continuing education is carried out by numerous bodies or institutions in the majority of the countries considered. This is particularly the case in Germany, the Netherlands, Belgium, the UK and Switzerland. In France, where the contents of the training are determined, there is also a large number of providers. Only in Norway most of the training is provided by the Norwegian Medical Association (Norske Legeforening) itself.

Apart from the UK, general practice CME is accredited in all the countries considered. In Switzerland, for example, CME points are awarded via the society of General Practitioners. In Norway, this is done by the Norwegian Medical Association or its subject-specific committees. In France, the “Agence Nationale du Développement Professionnel Continu” (ANDPC) is responsible, in Germany the federal state-specific “Landesärztekammern” and in Belgium the “Rijksinstituut voor Ziekte en Invaliditeitsverzekering”. In all countries included here, the individual or institutional accreditation of CME is rather formal and mainly based on various key data, such as topic, speaker and content.

### Funding and sponsorship

While in the UK the sponsoring of general practice CME by the pharmaceutical industry is generally regarded uncritically or as unproblematic, this is fundamentally different in the other countries considered. In the Netherlands and France, the sponsoring of medical training is prohibited and strictly controlled. In Norway, sponsorship by the pharmaceutical industry fundamentally precludes CME accreditation. In Switzerland, where financial support from industry is possible, at least two companies must participate. In Switzerland, sponsors officially have no influence on the content and course of the event or on the selection of speakers. Furthermore, participants must bear a reasonable share of the costs despite sponsorship. In Belgium, pharmaceutical sponsorship is only possible within a very restrictive framework. In Germany, directly and indirectly pharmaceutical-sponsored training courses for GPs are increasingly viewed critically, but they are still frequently offered and attended.

Independent of these developments, structures exist in the UK, Norway, Belgium and France that compensate financial expenses for general practice continuing medical education. In the UK, GPs receive lump sums depending on the region, which compensate for the costs of self-organised training. In Norway, most of the costs for GP training, including travel costs, are financed by the Norwegian Medical Association’s training fund. In Belgium, accredited GPs receive an annual lump-sum allowance for training costs. And in France, in addition to the costs paid for training events, doctors also receive a compensation payment for the loss of earnings incurred during the period of training. This is financed, among other things, by a compulsory levy on pharmaceutical companies based in France. GPs in the Netherlands and, in the case of non-sponsored events, in Switzerland and Germany largely finance their own CME.

### Evaluation

Except for the UK where unstructured feedback will usually suffice, CME courses for GPs are systematically evaluated in all the countries studied. In most cases, this is done by means of standardised questionnaires provided by the accrediting institutions or the organisers, which are filled out by the participants after the respective training event. In the vast majority of cases, the event is evaluated subjectively by the participants in terms of learning atmosphere, relevance and learning success. There is no objective assessment of learning and competence gains. In Switzerland and Norway, “success checks” at the end of training events are common. In Belgium, only the evaluation of e-learning is obligatory.

## Discussion

### Main findings

The comparison shows that none of the analysed countries has established a system of general practice specific CME that addresses all our predefined criteria - so there are no “magic bullets”. In all systems, the resources available for GP training are limited - be it in terms of time or economy. The way in which general practice continuing education takes place seems to be determined by medico-cultural traditions and the status of general practice in the prevailing health system.

### Strengths and limitations

The results of this narrative review are limited by the focus on north-western European countries. Also, we are aware that a narrative review can have gaps. To address this drawback, we have taken as structured an approach as possible and collected information along a matrix with the help of country experts. The project benefited greatly from this cooperation.

### Interpretation of findings

Regarding future developments, depending on the system, there are several fields of action that seem to be particularly worthy of discussion. Apart from France, there are no curriculum objectives for GPs in any of the countries studied. The development of a catalogue of learning objectives seems to make sense, which focuses on the maintenance and expansion of competences as well as the process of moving from CME to CPD.

Regarding the teaching formats used, the international comparison has shown that the classic formats - formal lectures - have a certain status in every system. Nevertheless, the importance of innovative teaching and learning formats, and here especially peer exchange, is increasing [6, 54]. In the shadow of the Corona pandemic, teaching formats have inevitably had to evolve and have experienced a push towards more digitalisation in all the countries considered. E-leaning and peer-led webinars have become more important. We know from implementation research that both peer exchange and feedback are important instruments for reflecting on one’s own professional activities and have measurable effects on performance [55]. Therefore, when evaluating GP training, the teaching format should also be considered more in the future and CME points should be awarded in a differentiated manner [56, 57].

Although already implemented in some countries, many countries hardly offer joint events for doctors in specialist training and practicing general practitioners. Yet this could be profitable for both groups. While the doctors in specialist training are still closer to their studies and approach topics relevant to general practice care with a critical curiosity, the GPs in private practice could contribute their professional expertise and ability for realistic reflection. At the same time, the networking of “young” and “old” colleagues could be promoted [56, 57].

In some countries we found a varied mix of GP training in terms of quantity and quality which does not lend itself to robust evaluation. In addition to innovative approaches, which in some cases meets curriculum requirements, there is still some content guided by different interests (e.g. from the pharmaceutical industry, or health policy prioritisations) that are, in the worst cases, inappropriate. A comprehensive learning platform which lists general practice CME courses and communicates their contents and learning objectives based on defined criteria, appears urgently advisable.

Regarding the sponsoring of CME events by the pharmaceutical industry, there is a corresponding awareness of the problem in all the countries considered [58]. However, most countries have developed and implemented different strategies to deal with possible influence. While pharma financed events in the Netherlands and Norway do not receive CME accreditation, in Germany, for example, the discussion about the influence of sponsorship on medical continuing education is somewhat stuck. Although efforts could be made to ensure that sponsorship is no longer allowed at all, at least for accredited CME events, obviously the current CME program in several countries only works because companies often finance or at least top up large parts of the speakers’ fees and room rents. To change this, separate funding is necessary.

Good teaching requires sufficient financial resources. Internationally, most of the countries examined here have implemented some ways to fund GP training. The discussion of which “pots” the necessary funds have to be taken from is outside the scope of this paper, although the international comparison suggests joint financing is valuable, by health insurance funds (and thus the insured), state institutions and the physicians themselves. From the insurers’ point of view, in turn, financial gains can be expected from greater rational use of diagnostics and therapy. Further, the recognition of GP training within working time also seems important. This is already being implemented in several countries.

The evaluation of CME for GPs has so far usually only been carried out on the basis of descriptive criteria, primarily aimed at subjective participant satisfaction. None of the countries compared used elaborate evaluation methodology. In future, scientifically based evaluations should focus on whether previously formulated learning objectives were achieved, competencies were imparted, further developed, or expanded and whether the performance of the participating physicians has improved further through participation in individual events or a longitudinal, curricular-based continuing education programme. To gain deeper insights, GPs could take a test after completing their CME training. The corresponding results could then form the basis for the further development of the system of continuing professional development. 

### Implications

Future CME programs for GPs face a variety of challenges. They should develop curriculum objectives, promote innovative teaching and learning formats, use synergies with specialist GP training, create comprehensive learning platforms, establish clear rules for sponsorship, develop new financing models, and scientifically evaluate CME training. Especially in view of European cooperation (for instance in border areas) and EU-wide freedom of movement, cross-EU CME programs should be promoted in the future. Also, in view of the limited financial resources, it will be important in future to orient CME in such a way that questions about the efficiency and quality of primary medical care are given greater attention. The current pandemic will further strengthen this process. Moreover, the experience of the Corona pandemic shows us that cooperation between primary care providers and health authorities can be incredibly important. Forecasts of an increase in pandemics underpin the importance of appropriate training content in CME.

## Conclusions

Although the analysed models can be considered as precursors in different dimensions, there is no model that fully meets the current requirements of GP specific CME. The country-specific approaches described therefore offer selective stimulus for future developments, but each leaves more or less room for further development.

## Data Availability

Matrixes with country-specific information are available on request (christin.loeffler@med.uni-rostock.de).

## Abbreviations

ANDPC: Agence Nationale du Développement Professionnel Continu France
CME: Continuing Medical Education
CMG: Collège de Médecine Générale, France
CNP: National Professional Councils, France
CPD: Continuing Professional Development
DMPs: Disease Management Programmes
GAIA: Gemeenschappelijke Accreditatie Internet Applicatie, The Netherlands
GMC: General Medical Council, UK
GPs: General Practitioners
MVZ: Medical Care Centres, Germany
NHS: National Health Service, UK
SGAIM: Swiss Society of General Internal Medicine
SIWF: Swiss Institute for CME

## References

[1] Opstelten W, Cals JWL, van der Horst HE (2020). General practice: continuously developing. Ned Tijdschr Geneeskd.

[2] Park S, Abrams R, Wong G (2019). Reorganisation of general practice: be careful what you wish for. Br J Gen Pract.

[3] Barnett K, Mercer SW, Norbury M (2012). Epidemiology of multimorbidity and implications for health care, research, and medical education: a cross-sectional study. Lancet.

[4] O'Riordan M, Dahinden A, Aktürk Z (2011). Dealing with uncertainty in general practice: an essential skill for the general practitioner. Qual Prim Care.

[5] Davis M, Forrest K (2008). How to teach continuing medical education.

[6] Cervero RM, Gaines JK (2015). The impact of CME on physician performance and patient health outcomes: an updated synthesis of systematic reviews. J Contin Educ Heal Prof.

[7] Fereday J, Muir-Cochrane E. Demonstrating rigor using thematic analysis: a hybrid approach of inductive and deductive coding and theme development. Int J Qual Methods. 2006;5(1):80–92. 10.1177/160940690600500107.

[8] Boyatzis R (1998). Transforming qualitative information: thematic analysis and code development.

[9] Kaiser R (2014). Qualitative Experteninterviews: Konzeptionelle Grundlagen und praktische Durchführung [qualitative expert interviews: conceptual foundations and practical implementation].

[10] Bogner A, Littig B, Menz W (2014). Interviews mit Experten: Eine praxisorientierte Einführung [interviews with experts: a practice-oriented introduction].

[11] Gläser J, Laudel G (2010). Experteninterviews und qualitative Inhaltsanalyse: als Instrumente rekonstruierender Untersuchungen [expert interviews and qualitative content analysis: as instruments of reconstructive studies].

[12] Przyborski A, Wohlrab-Sahr M (2013). Qualitative Sozialforschung: Ein Arbeitsbuch [qualitative social research: a workbook].

[13] General Medical Council (UK). Continuing professional development: Guidance for all doctors [internet]. England and Wales, and Scottland. Available from: https://www.gmc-uk.org/-/media/documents/cpd-guidance-for-all-doctors-0316_pdf-56438625.pdf. [Cited 2021 Oct 25].

[14] General Medical Council (UK). Guidance on supporting information for appraisal and revalidation [internet]. England and Wales, and Scottland. Available from: https://www.gmc-uk.org/registration-and-licensing/managing-your-registration/revalidation/guidance-on-supporting-information-for-appraisal-and-revalidation. [Cited 2021 Oct 25].

[15] Royal College of GPs (UK). Guide to supporting information for appraisal and revalidation [internet]. England. Available from: http://www.gpappraisals.uk/uploads/4/5/8/5/4585426/rcgp-guide-to-supporting-information-2018.pdf. [Cited 2021 Oct 25].

[16] National Health Service England. Responsible Officers. [internet]. England. Available from: https://www.england.nhs.uk/medical-revalidation/ro/. [Cited 2021 Oct 25].

[17] National Audit Office Report (England). Prescribing Costs in Primary Care [internet]. England. Available from: https://www.nao.org.uk/report/prescribing-costs-in-primary-care/. [Cited 2021 Oct 25].

[18] Tzortziou Brown V, McCartney M, Heneghan C (2020). Appraisal and revalidation for UK doctors—time to assess the evidence. BMJ..

[19] Cate OT (2007). Medical education in the Netherlands. Medical Teacher..

[20] Hoff RG, Frenkel J, Imhof SM, Cate OT (2018). Flexibility in postgraduate medical training in the Netherlands. Acad Med.

[21] Cate OT, Carraccio C (2019). Envisioning a true continuum of competency-based medical education, training, and practice. Acad Med.

[22] Hillen HFP (2010). Quality assurance of medical education in the Netherlands: programme or systems accreditation?. Tijdschrift voor Medisch Onderwijs.

[23] Jaarsma D, Scherpbier A, Van Der Vleuten C, Cate OT (2013). Stimulating medical education research in the Netherlands. Medical Teacher.

[24] European Union of General Practitioners/ Family Physicians. General practise in Norway – increasing its popularity? A Some important issues in general practice/family medicine in Norway, [internet]. Available from: https://www.uemo.eu/norway. [Cited 2022 Sept 5].

[25] Bjorvatn A. Wage Differentials Among General Practitioners in Norway. iHEA 2007 6th World Congress: Explorations in Health Economics Paper. [internet]. Available from: https://ssrn.com/abstract=993731. [Cited 2022 Sept 5].

[26] Helsedirektoratet (Norway). Allmennmedisin [General Practice], [internet]. Oslo. Available from: https://www.helsedirektoratet.no/tema/autorisasjon-og-spesialistutdanning/spesialistutdanning-for-leger/allmennmedisin. [Cited 2021 Oct 25].

[27] Helsedirektoratet (Norway). FREMTIDENS LEGESPESIALISTER Spesialitetsstruktur og -innhold i samfunnsog allmennmedisin [THE MEDICAL SPECIALISTS OF THE FUTURE Specialty structure and content in community and general medicine], [internet]. Oslo. Available from: https://www.helsedirektoratet.no/rapporter/fremtidens-legespesialister/Fremtidens%20legespesialister%20%E2%80%93%20spesialitetsstruktur%20og%20innhold%20i%20samfunns%20og%20allmennmedisin.pdf/_/attachment/inline/a8e33fcc-d8d3-4b56-b861-0af291e76ab9:c8bbadc73a6839f2e337f8b8abfeda6402d2ff3e/Fremtidens%20legespesialister%20%E2%80%93%20spesialitetsstruktur%20og%20innhold%20i%20samfunns%20og%20allmennmedisin.pdf. [Cited 2021 Oct 25].

[28] Garattini L, Gritti S, De Compadri P, Casadei G (2010). Continuing medical education in six European countries: a comparative analysis. Health Policy.

[29] Sánchez-Sagrado T (2017). La atención primaria en Bélgica [Primary care in Belgium]. Semergen..

[30] VanNieuwenborg L, Goossens M, De Lepeleire J, Schoenmakers B (2016). Continuing medical education for general practitioners: a practice format. Postgrad Med J.

[31] Wet inzake de kwaliteitsvolle praktijkvoering in de gezondheidszorg [Health Care Quality Practice Act, Belgium], [internet]. Brussels. Available from: http://www.ejustice.just.fgov.be/eli/wet/2019/04/22/2019041141/justel. [Cited 2021 Oct 25].

[32] Koninklijk besluit tot uitvoering van de wet betreffende de verplichte verzekering voor geneeskundige verzorging en uitkeringen [Royal Decree implementing the Act on compulsory insurance for medical care and benefits, Belgium] Art. 122octies/1–8, Royal Decree 3, July 1996 [internet]. Brussels. Available from: http://www.ejustice.just.fgov.be/eli/besluit/1996/07/03/1996022344/justel. [Cited 2021 Oct 25].

[33] Rijksinstituut voor ziekte- en invaliditeitsverzekering (Belgium). Accreditering [Accreditation], [internet]. Brussels. Available from: https://www.riziv.fgov.be/nl/toepassingen/Paginas/accreditering-webtoepassing.aspx. [Cited 2021 Oct 25].

[34] Koninklijk besluit tot instelling van een financiering voor lokale kwaliteitsgroepen [Royal Decree establishing funding for local quality groups], Royal Decree 12, December 2012, [internet]. Brussels. Available from: http://www.ejustice.just.fgov.be/eli/besluit/2012/12/12/2012022477/justel. [Cited 2021 Oct 25].

[35] Bundesärztekammer (Germany). (Muster-)Fortbildungsordnung der Bundesärztekammer [(Model) Continuing Education Regulations of the German Medical Association], [internet]. Berlin. Available from: https://www.bundesaerztekammer.de/fileadmin/user_upload/downloads/_Muster-_Fortbildungsordnung_29052013.pdf. [Cited 2021 Sept 23].

[36] Deutsche Gesellschaft für Allgemeinmedizin und Familienmedizin (Germany). Professionelles Lernen von Hausärzten – ein Leben lang. Stellungnahme zur ärztlichen Fortbildung [Professional learning of GPs - for life. Statement on continuing medical education], [internet]. Berlin. Available from: https://www.degam.de/files/Inhalte/Degam-Inhalte/Sektionen_und_Arbeitsgruppen/Sektion_Fortbildung/Startseite/DEGAM-Fortbildungspapier2009-Langversion-Hintergrund-Papier.pdf. [Cited 2021 Nov 17].

[37] Egidi G, Biesewig-Siebenmorgen J, Schmiemann G (2011). 5 Jahre Akademie für hausärztliche Fortbildung Bremen – Rückblick und Perspektiven [5 years academy for continuing education in family medicine Bremen - review and perspectives]. Z Allg Med.

[38] Sturm D (2017). Fortbildung für Hausärzte oder hausärztliche Fortbildung? [Continuing education for GPs or GP training?]. Z Allg Med..

[39] Ruf D, Berner MM, Kriston L, Maier I, Härter M (2008). Hausärzte online: Gute Voraussetzungen, aber geringe Nutzung des Internets zur Fortbildung [GPs online: Good conditions, but low use of the internet for CME]. Zeitschrift für Evidenz, Fortbildung und Qualität im Gesundheitswesen.

[40] Lempert T, Schott G, Köbberling J (2019). Fortbildungen: Keine Punkte bei sponsoring [CME: no credits for sponsoring]. Dtsch Arztebl.

[41] Cartier T, Senn N, Cornuz J, et al. Switzerland. In: Kringos DS, Boerma WGW, Hutchinson A, et al., eds. Building primary care in a changing Europe. Copenhagen, Denmark: European Observatory on Health Systems and Policies; 2015:275–284, [internet]. Copenhagen. Available from: https://www.euro.who.int/__data/assets/pdf_file/0011/277940/Building-primary-care-changing-Europe-case-studies.pdf. [Cited 2021 Nov 26].

[42] Bundesgesetz über die universitären Medizinalberufe, 2006. [Federal Act on the University Medical Professions, Switzerland], [internet]. Bern. Available from: https://www.admin.ch/opc/de/classified-compilation/20040265/index.html. [Cited 2021 Oct 25].

[43] Schweizer Institut für ärztliche Fort- und Weiterbildung, SIWF, [internet]. Bern. Available from: https://www.siwf.ch/fortbildung/fortbildungsprogramme.cfm. [Cited 2021 Oct 25].

[44] Swiss Society of General Internal Medicine, SGAIM, [internet]. Bern. Available from: https://www.sgaim.ch/de/fortbildung/fortbildungsnachweis.html. [Cited 2021 Oct 25].

[45] Ministère des Solidarités et de la Santé (France). Médecin généraliste [General Practice], [internet]. Paris. Available from: https://solidarites-sante.gouv.fr/metiers-et-concours/les-metiers-de-la-sante/les-fiches-metiers/article/medecin-generaliste. [Cited 2021 Oct 25].

[46] Agence nationale du Développement Professionnel Continu (DPC, France). Mon DPC - Document de traçabilité [My CME - Tracking document], [internet]. Le Kremlin Bicetre Cedex. Available from: https://www.agencedpc.fr/le-dpc/mon-dpc-document-de tra%C3%A7abilit%C3%A9?__cf_chl_jschl_tk__=pmd_7_rnHZNFbOdLNh6l.zpjbpEEKfCzexo4UQUKULWG5EA-1635320828-0-gqNtZGzNApCjcnBszQnR. [Cited 2021 Oct 26].

[47] Collège de la Médecine Générale (France). Qu’est ce que le DPC? [What is CPD?], [internet]. Paris. Available from: https://lecmg.fr/le-developpement-professionnel-continu/. [Cited 2021 Oct 25].

[48] Collège National des Généralistes Enseignants. Le développement professionnel continu [Continuing professional development], [internet]. Paris. Available from: https://www.cnge.fr/la_formation/le_developpement_professionnel_continu/. [Cited 2021 Oct 25].

[49] Agence nationale du Développement Professionnel Continu (DPC, France). Fiches de cadrage relatives aux orientations pluriannuelles prioritaires 2020–2022 [Framework sheets for the multiannual priority guidelines 2020–2022], [internet]. Le Kremlin Bicetre Cedex. Available from: https://www.agencedpc.fr/sites/default/files/images/article/tableau_orientations2020_2022.pdf. [Cited 2021 Oct 26].

[50] Société de Formation Thérapeutique du Généraliste (France). Fiches d’orientations pluriannuelles prioritaires de DPC pour les professions/ spécialités [Multi-annual priority CME guidance sheets for CME for professions/specialties], [internet]. Paris. Available from: https://www.sftg.eu/media/agence_nationale_du_dpc_op_mg.pdf. [Cited 2021 Oct 25].

[51] Haute Autorité de Santé. Développement professionnel continu (DPC) [Continuing Professional Development (CPD)], [internet]. Saint-Denis La Plaine Cedex. Available from: https://www.has-sante.fr/jcms/p_3019317/fr/demarche-et-methodes-de-dpc. [Cited 2021 Oct 25].

[52] Maisonneuve H (2014). How France messed up CPD/CME for healthcare professionals, Editorial. BMJ.

[53] Bertrand D, Bouet P (2020). Développement professionnel continu (DPC) et émergence de la recertification en France. Évolution législative et commentaires [Continuing professional development and recertification process in France]. Bull Acad Natl Med.

[54] Cantillon P, Jones R (1999). Does continuing medical education in general practice make a difference?. BMJ..

[55] Lorthios-Guilledroit A, Richard L, Filiatrault J (2018). Factors associated with the implementation of community-based peer-led health promotion programs: a scoping review. Eval Program Plann.

[56] Morgan JHC (2003). New approaches to training general practitioners. Postgrad Med J.

[57] Fraser SW, Greenhalgh T (2001). Coping with complexity: educating for capability. BMJ..

[58] Lieb K, Brandtönies S (2010). A survey of German physicians in private practice about contacts with pharmaceutical sales representatives. Dtsch Arztebl Int.

